# Anticancer Effect of the Ethyl Acetate Fraction from *Orostachys japonicus* on MDA-MB-231 Human Breast Cancer Cells through Extensive Induction of Apoptosis, Cell Cycle Arrest, and Antimetastasis

**DOI:** 10.1155/2019/8951510

**Published:** 2019-11-06

**Authors:** Ji-Hye Kwon, Jae-Hyeon Kim, Deok-Seon Ryu, Hyun-Ji Lee, Dong-Seok Lee

**Affiliations:** ^1^Department of Biomedical Laboratory Science, Inje University, 197 Inje-ro, Gimhae 50834, Gyeongnam, Republic of Korea; ^2^Department of Biomedical Laboratory Science, Soonchunhyang University, Asan 31538, Chungnam, Republic of Korea

## Abstract

The antibreast cancer activities of the ethyl acetate fraction from *Orostachys japonicus* (OJEF) were investigated in MDA-MB-231 human breast cancer cells through WST assay, DAPI staining, flow cytometry analysis, and western blotting. OJEF effectively inhibited MDA-MB-231 cells by inducing apoptosis via intrinsic, extrinsic, and endoplasmic reticulum (ER) stress response pathways, cell cycle arrest at the G1/S phase, and antimetastasis including inhibition of tight junction, adherens junction, invasion, and migration. The MAPK family-mediated upstream signal transduction through p-p38 and p-ERK was considered to affect the downstream signal transduction including induction of apoptosis, cell cycle arrest, and antimetastasis. In conclusion, we executed an integrated study on the anticancer activities of OJEF, which extensively induced apoptosis, cell cycle arrest, and antimetastasis in estrogen-independent MDA-MB-231 human breast cancer cells known to be liable to metastasize.

## 1. Introduction

According to the latest data, cancer is the leading cause of mortality in Korea. Among all cancers, breast cancer is the second main cause of cancer-related death in women worldwide today [1]. Usually, cancer is treated with surgery, radiotherapy, immunotherapy, or chemotherapy. Most current chemotherapies are combinations of chemical substances with low or no selectivity towards cancer cells, and they are usually toxic to both cancer and normal cells. In recent years, many studies have been conducted to find new anticancer drugs that are only effective to cancer cells to avoid causing harm to patients. Researchers have recently moved actively towards discovering biologically active materials with anticancer activity in medicinal herbs, as these could be harmless than existing anticancer drugs. *Orostachys japonicus* is known as a herbaceous plant for its potent antiinflammatory, antifebrile, hemostatic, antidotal, and particularly anticancer activities [2–6]. Abnormal apoptosis is known to cause cancer and degenerative diseases. Therefore, recovering normal apoptosis in cancer cells has been considered a key indicator of the anticancer activity of potential remedy substances [7]. When apoptosis occurs in a cell, phosphatidylserine (PS) becomes exposed on the outer membrane, impeding the antiapoptotic protein B-cell lymphoma-2 (bcl-2) and activating the apoptosis-induced protein, bax [8]. As a result, apoptosis-causing proteins called caspases are activated by the release of cytochrome c from the mitochondria [9–18]. Subsequent drastic changes occur in the nucleus, including DNA fragmentation through the activation of endonucleases, chromatin condensation, nuclear envelope breakdown, and nucleus vacuolation [8, 19]. Furthermore, since cancer cells continue to proliferate uncontrollably without maintaining normal proliferation, the cell cycle arrest is another definite indicator of anticancer activity. Cell division is divided into the G1 phase, the synthetic S phase, the G2 phase, and the M phase for mitosis. There are 3 checkpoints for problem-free cell division and smooth transition between the phases. The first is the restriction point in the late G1 stage, at which the cell admit entry of cell cycle and duplication of chromosome. The second checkpoint is the G2/M transition, at which the control system starts the early mitotic events, leading to chromosome alignment on the spindle in the metaphase. The third checkpoint is the metaphase/anaphase transition, at which the control system prompts sister-chromatid separation, causing the completion of mitosis and cytokinesis [20]. Moreover, the level of migration, invasion, and metastasis is another indicator of anticancer activity. The largest benefit of compounds with anticancer activity is cancer prevention, and after cancer forms, anticancer compounds suppress the proliferation of cancer cells and invasion and migration into other organs [9, 21]. In this regard, dysregulated intercellular adhesion between cells is related to carcinogenesis, accelerated invasion, increased migration, and induction of metastasis [10]. The invasion of the cancerous cells involves the process of dismantling the extracellular matrix (ECM) and the basement membrane with proteolytic enzymes known as matrix metalloproteinases (MMPs), and cancer cells then migrate through the decomposed substrates [10, 11]. In addition, there are three types of intercellular adhesion junctions such as tight junction, adherens junction, and desmosome junction. Claudin, occludin, and zo-1 are known as tight junction-related proteins, and cadherin and *β*-catenin are known as adherens junction-related proteins [12, 13]. Especially, *β*-catenin is a transcription factor that engages in intracellular Wnt signal transduction in addition to its role of connecting cadherin to the cellular skeleton. *β*-Catenin is known to activate Wnt signal transduction and induce cell transformation [14, 15]. There are currently 26 reports concerning *O. japonicus* indexed on PubMed, with only 10 related to anticancer activities [4–7, 19–21]. To date, there has been no study conducted in breast cancer cells, and studies on other cancers were only restricted to apoptosis induction and/or cell cycle arrest without studying antimetastasis. Furthermore, there are currently about 1,000 papers about antibreast cancer activities of biologically active substances from other herbaceous plants, and these reports were also mainly confined to apoptosis or cell cycle arrest. In this study, we explored the inhibitory activity of the ethyl acetate fraction from *O. japonicus* (OJEF) in MDA-MB-231 human breast cancer cells; we examined antimetastasis as well as apoptosis and cell cycle arrest; thus, this study is further advanced and differentiated from previous studies. Therefore, the purpose of this work was to systematically establish the anticancer activities of OJEF in estrogen-independent MDA-MB-231 cells known to be prone to metastasize by investigating the molecular mechanisms on overall induction of apoptosis, cell cycle arrest, and antimetastasis including inhibition of tight junction, adherens junction, invasion, and migration.

## 2. Materials and Methods

### 2.1. Preparation of OJEF

The OJEF was prepared in our laboratory using a simply changed procedure described previously [2–5].

### 2.2. Cell Line and Reagents

MDA-MB-231 cells (human breast cancer cells, KCLB No. 30026) were obtained from the Korean Cell Line Bank (KCLB, Seoul, Korea). All other reagents in this study were of the highest grade or analytical grade [2–6].

### 2.3. Cell Culture and Treatment

The MDA-MB-231 cells were cultured in DMEM medium fortified with 10% heat-inactivated fetal bovine serum (FBS) and 1% penicillin/streptomycin and incubated at 37°C in a 5% CO_2_ humidified atmosphere incubator until they reached confluence. The cells were subcultured every 4 to 6 days at 1 : 5 split ratios, and the growth medium was replaced every 2 days. Cells at approximately 80–90% confluency were used in the experiments.

### 2.4. Cell Viability Assay

Cell viability was assessed by a EZ-CyTox enhanced cell viability assay kit (DoGEN Life Science Genetic Engineering, Daeil Lab Service Co., Ltd, Korea) according to manufacturer's instructions. In brief, cells (6 × 10^5^ cells/well) were seeded into well plates and incubated at 37°C with 5% CO_2_ for 24 h and then treated with varying concentrations of *O. japonicus* or in combination for 12  and 24 h. After incubation, 10 *μ*L of WST (2-(2-methoxy-4-nitrophenyl)-3-(4-nitrophenyl)-5-(2,4-disulfophenyl)-2H-tetrazolium, water-soluble tetrazolium salts) was added and then cultured for 2 h at the same condition. Thereafter, absorbance was directly measured at 450 nm with a microplate reader (Synergy HT, DI BIOTEK, USA) in the dark.

### 2.5. Nuclear Staining with 4′,6-Diamidino-2-Phenylindole (DAPI)

Nuclear staining with DAPI was performed using slightly modified methods described previously [3–6]. Harvested MDA-MB-231 cells were washed once with phosphate-buffered saline (PBS; 2.7 mM KCl, 10 mM Na_2_HPO_4_, 137 mM NaCl, pH 7.4) and then put back into PBS with 0.1% Triton X, and left for 10 min on ice. After centrifugation, cells were suspended again in 4% PBS-buffered paraformaldehyde solution including DAPI (Vector Laboratories, CA, USA). An aliquot (10 *µ*L) of this sample was put onto a slide glass, and the forms of the cells' nuclei were examined using a laser confocal fluorescence microscope (LSM510 Meta, Carl Zeiss, Jena, Germany), at 350 nm excitation wavelength.

### 2.6. Apoptosis Assay

Apoptosis in the MDA-MB-231 cells (6 × 10^5^ cells/mL in a 12-well plate) was evaluated by annexin V-fluorescein isothiocyanate (annexin V-FITC) and propidium iodide (PI) staining by using the BD Pharmingen Annexin V-FITC Apoptosis Detection Kit I (Becton Dickinson Biosciences, USA), according to the manufacturer's instructions [2–6]. MDA-MB-231 cells (4 × 10^5^ cells/mL in a 24-well plate) were mixed with different concentrations of the OJEF for 12 h and then collected by centrifugation at 300 ×g. After centrifugation, the pellets were rinsed twice with cold PBS and resuspended in 100 *μ*L of 1x binding buffer (2.5 mM CaCl_2_, 140 mM NaCl, 10 mM HEPES/NaOH, pH 7.4). The cells were placed with 5 *μ*L of annexin V-FITC and 5 *μ*L of PI at 20°C for 15 min in the dark. And then, 400 *μ*L of 1x binding buffer was put into each of the tube and the cells were analyzed immediately by FACSCalibur flow cytometry (Becton Dickinson, NJ, USA).

### 2.7. Cell Cycle Analysis

The cell cycle phase was measured by DNA fragment staining using the cell cycle phase determination kit (Cayman Chemical, Ann Arbor, MI, USA) [2–6]. MDA-MB-231 (4 × 10^5^ cells/mL in a 24-well plate) were added to different concentrations of the OJEF for 12 h and then collected. After centrifugation, the precipitates were washed and resuspended in cell-based assay buffer. The cells were fixed and permeated by treating 1 mL of a fixative to each tube for around 2 h. After centrifugation, the fixatives were removed and the cell pellets were put into 500 *μ*L of a staining solution (200 *μ*L of RNase and 200 *μ*L of PI), followed by leaving for 30 min at 20°C in the dark. Then, the cells were examined immediately by FACSCalibur flow cytometry.

### 2.8. Wound Healing Assay

The MDA-MB-231 cells were seeded at a concentration of 6 × 10^5^ cells/mL into a 6-well plate for cell culture, stabilized for 24 h, and allowed to grow until the 90% confluency. The media was aspirated and replaced with SFM containing different concentrations (0, 0.1% DMSO, 10, 20, 40, or 60 *μ*g/mL) of OJEF. They were artificially scraped in a straight line using sterilized 200 *μ*L pipette tip in the middle of the well (0 h). The level of wound healing was assayed using a phase difference microscope at a 40x magnification. The rate of migration towards the center of the wound was pictured at 0 and 24 h, respectively, after treatment with various concentrations of OJEF. The distances of migrating cells were measured from picture, and the distance of each measurement was calculated [1, 9].

### 2.9. Western Blotting Analysis

Western blotting analysis was conducted using slightly modified methods described previously [2–6]. The MDA-MB-231 cells were treated with the OJEF, washed twice with ice-cold PBS, and collected using a cell scraper. The cells were then precipitated by centrifugation, the pellets were suspended again in lysis buffer on ice for 1 h, and the cell debris were eliminated by centrifugation at 10,000 ×g for 10 min. Protein concentrations were analyzed using the Bicinchoninic acid (BCA) Protein Assay Kit (Thermo Scientific, IL, USA). Equal amounts of protein were combined with 2x Laemmli loading buffer and heated previously at 95°C for 5 min. The samples were electrophoresed on 10–15% sodium dodecyl sulfate-polyacrylamide gels and moved onto a polyvinylidene difluoride membrane for 1 h by using a semidry transfer system (Bio-Rad, CA, USA). The membrane was protected with 5% nonfat milk in PBS including 0.1% Tween 20 (PBST) for 2 h at 4°C and then incubated overnight with primary antibodies. After hybridization with primary antibodies, the membranes were cleansed for 5 min with PBST, 3 times. Subsequently, the membranes were placed with HRP-secondary antibody for 2 h at 4°C and rinsed for 5 min with PBST, 3 times. The mark of the membranes was generated by using a western blotting luminal reagent (Santa Cruz, CA, USA).

## 3. Results

### 3.1. Effect of OJEF on Cell Viability

**Figure 1:**
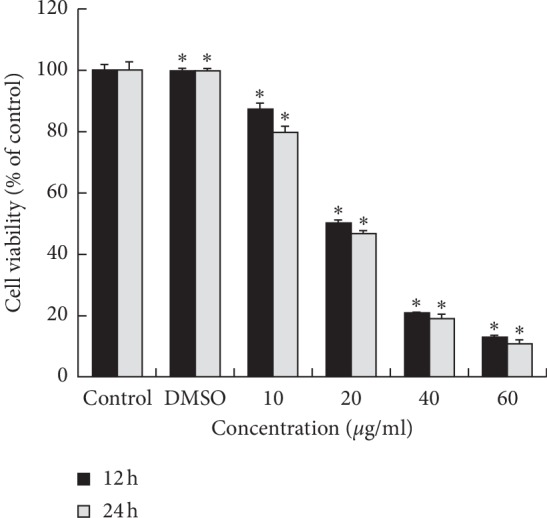
Effect of OJEF on cell viability for 12 and 24 h in MDA-MB-231 cells. The cells were treated with varying concentrations (0, 0.1% DMSO, 10, 20, 40, or 60 *μ*g/mL) of OJEF for 12 and 24 h. Cell viability was assessed using WST assay. The values are expressed as the means ± S.D. (*n* = 5). Values of ^*∗*^*p* < 0.001 were considered statistically significant.

WST assay was performed to assess the effect of OJEF on the survival of MDA-MB-231 human breast cancer cells. After cells were treated with OJEF at various concentrations for 12 and 24 h, cell viability decreased in a dose-dependent manner (Figure 1). The OJEF exerted no effect on survival and proliferation of normal macrophage cells across a range of doses in a previous report [16].

### 3.2. Induction of Apoptosis by OJEF

**Figure 2:**
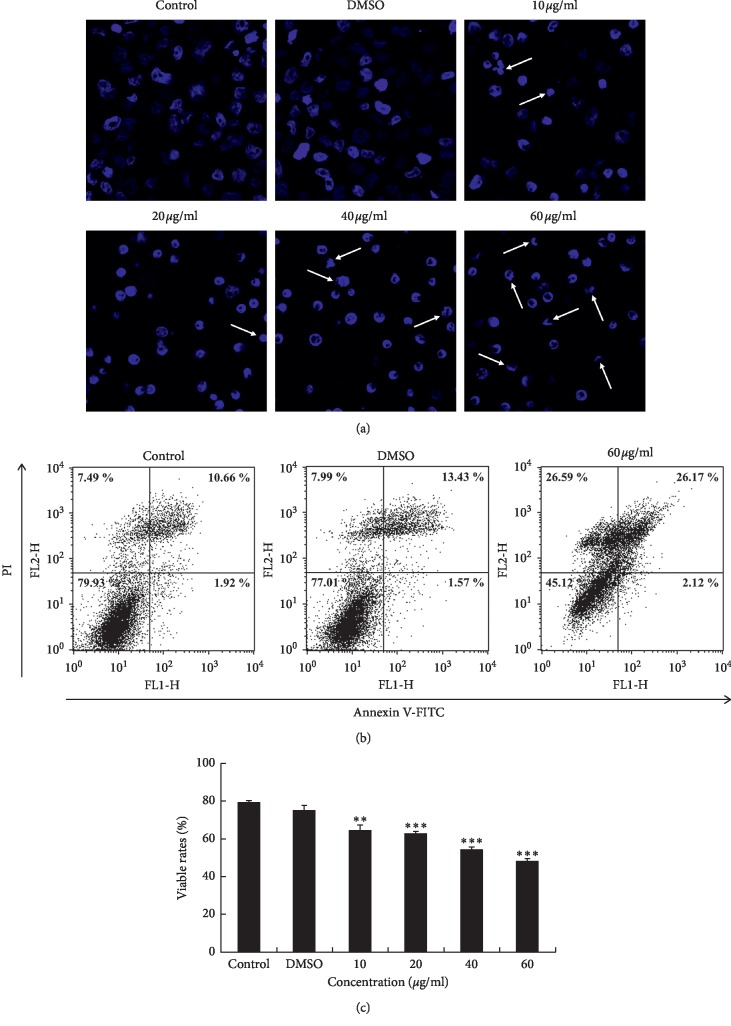
DAPI staining and flow cytometry analysis of apoptotic death of MDA-MB-231 cells. (a) Induction of apoptosis in MDA-MB-231 cells. The cells were treated with OJEF (0, 0.1% DMSO, 10, 20, 40, or 60 *μ*g/mL) for 24 h and then stained with the DNA-specific fluorochrome DAPI. Apoptotic bodies are indicated by white arrows. (b) and (c) denote flow cytometry analysis of the apoptotic death of MDA-MB-231 cells. (b) Dot plots show the apoptotic death of MDA-MB-231 cells treated with OJEF (0, 0.1% DMSO, or 60 *μ*g/mL) for 12 h. The cells were stained with monoclonal antibodies against Annexin V-FITC and PI. Annexin−/PI− (LL), viable cells; Annexin+/PI− (LR), cells undergoing apoptosis (early apoptosis); Annexin+/PI+ (UR), cells in end-stage apoptosis (late apoptosis) or already dead. LL, lower left; LR, lower right; UR, upper right. Three experiments showed similar results. (c) Bars indicate the percentage of viable cells treated with OJEF (0, 0.1% DMSO, 10, 20, 40, or 60 *μ*g/mL) for 12 h The values are expressed as the means ± S.D. (*n* = 3). Values of ^*∗*^*p* < 0.05, ^*∗∗*^*p* < 0.01, ^*∗∗∗*^*p* < 0.001 were considered statistically meaningful.

Staining with DAPI showed condensed chromatin, fragmented nuclei, and apoptotic bodies in OJEF-treated cells. The percentage of apoptotic bodies increased in a dose-dependent manner (Figure 2(a)). These morphological changes represent that OJEF induces apoptosis in MDA-MB-231 cells [7]. In the early phases of apoptosis, PS is exposed to the outer membrane, which is considered an early indicator of apoptosis. Annexin V is a protein that specifically binds to PS as a Ca^2+^-related phospholipid-binding protein. Using this method, exposed PS can be found. Simultaneously, damaged DNA was coloured with PI. In other words, apparent apoptosis phases can be discerned by combining Annexin V and PI staining. The lower left quadrants in Figure 2(b) depict a viable normal cell group without damage. The lower right quadrants exhibit cells undergoing early apoptosis with PS exposed to the outer layer of the membrane and DNA not stained by PI. The upper right quadrants display late apoptosis [5]. The total apoptotic rate (28.29%) of treated cells was greater than that of the control (12.58%). Overall, these results indicate that OJEF treatment increased the total apoptotic rate (Figures 2(b) and 2(c)).

### 3.3. Induction of Cell Cycle Arrest by OJEF

**Figure 3:**
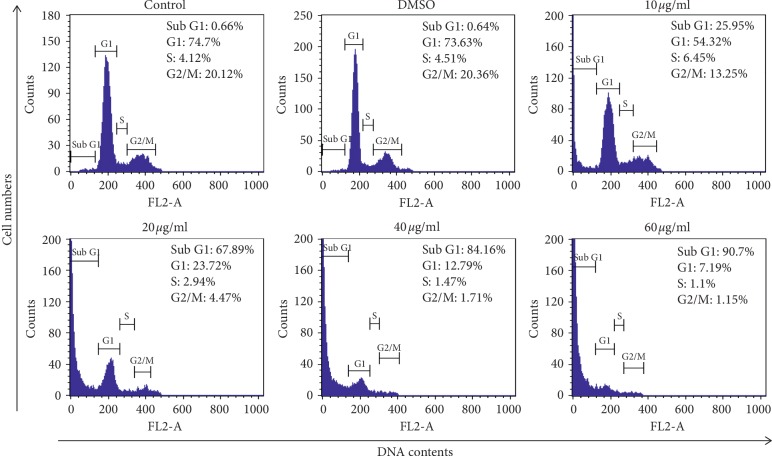
Flow cytometry analysis of cell cycle distribution of MDA-MB-231 cells. The cells were treated with OJEF (0, 0.1% DMSO, 10, 20, 40, or 60 *μ*g/mL) for 12 h. Histograms represent sub-G1, G1, S, and G2/M phases of MDA-MB-231 cells. The results were expressed as percentage of total treated cells. Three experiments showed similar results.

FACS analysis was performed to analyze the cell cycle in cancer cells using PI staining. As shown in Figure 3, the DNA contents of PI-stained MDA-MB-231 cells were established by flow cytometry. The fragmented DNA, evidence of apoptosis, appeared on the left side of the G1 peak of the cell cycle. Apoptosis is confirmed by finding the presence of this sub-G1 peak, which was increased in an OJEF dose-dependent manner.

### 3.4. Inhibition of Cell Migration by OJEF

**Figure 4:**
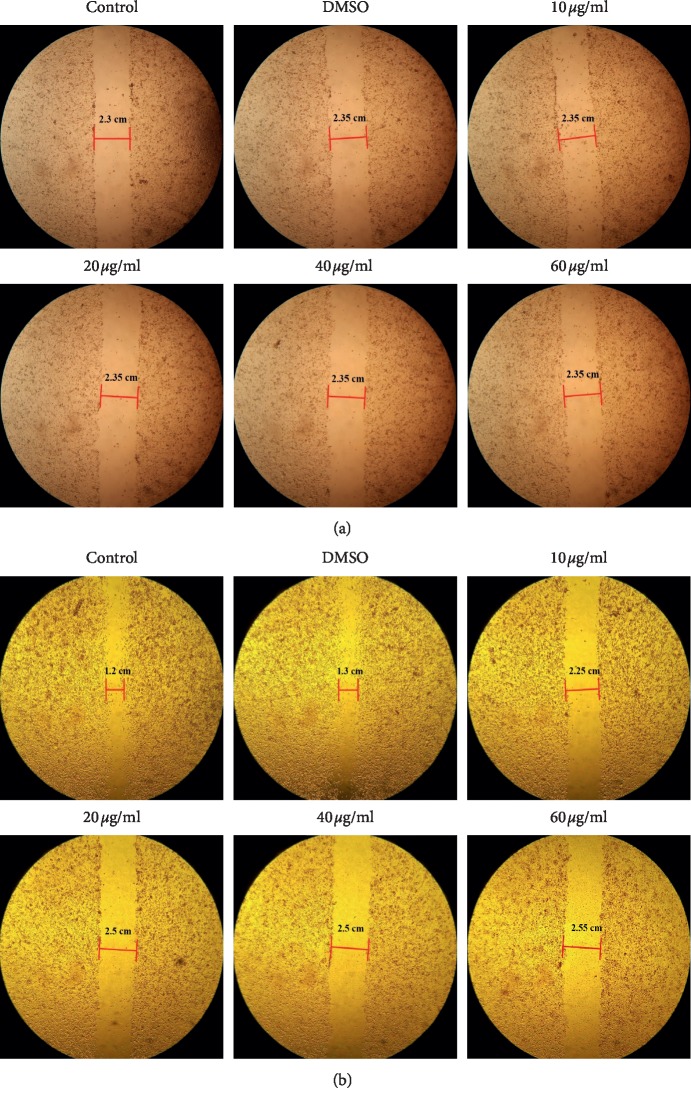
Inhibitory effect of OJEF on the migration of MDA-MB-231 cells. The cells were incubated in the medium treated with OJEF (0, 0.1% DMSO, 10, 20, 40, or 60 *μ*g/mL). (a) Results of in vitro wound healing assay for 0 h. (b) Results of in vitro wound healing assay for 24 h. Three experiments showed similar results.

To examine the antimetastasis effect of OJEF, the wound healing assay was conducted [1, 9]. The area of the wound was measured at two points in each group, and the distance between bars marked with red color was compared.  Figure 4(a) denotes the control group for 0 h.  Figure 4(b) represents definite inhibition of migration by OJEF of different concentrations after 24 h.

### 3.5. Effect of OJEF on the Expression of Apoptosis-Related Proteins

**Figure 5:**
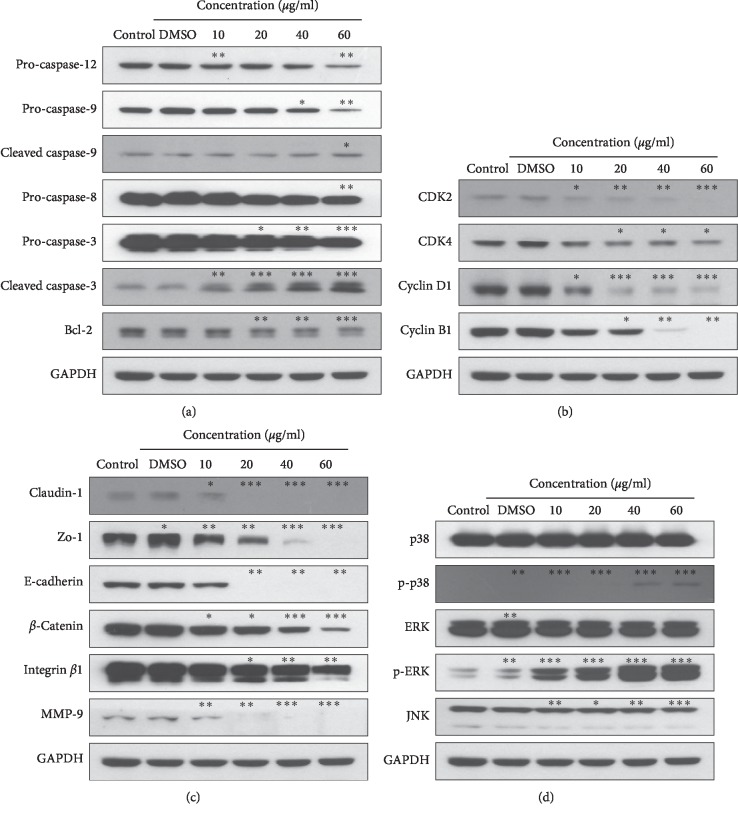
Effect of OJEF on the protein levels of (a) bcl-2, pro-caspase-3, -8, -9, and -12, and cleaved caspase-3 and -9, (b) CDK2, CDK4, cyclin D1, and cyclin B1, (c) claudin-1, zo-1, E-cadherin, *β*-catenin, integrin *β*1, and MMP-9, and (d) MAPK family in MDA-MB-231 human breast cancer cells. The cells were treated with OJEF (0, 0.1% DMSO, 10, 20, 40, or 60 *µ*g/mL) for 12 h. Expression of the indicated proteins was examined by western blotting. The density of bands was quantitated. GAPDH was used as an internal control. Band intensities were measured by densitometry in three separate experiments with similar results. The values are indicated as the means ± S.D. (*n* = 3). Values of ^*∗*^*p* < 0.05, ^*∗∗*^*p* < 0.01, and ^*∗∗∗*^*p* < 0.001 were considered statistically meaningful.

We performed western blotting to confirm the apoptosis results obtained by FACS analysis at the protein level. As shown in Figure 5(a), the expression levels of pro-caspase-3, -8, -9, and -12 and bcl-2 in MDA-MB-231 cells after treatment with each concentration of OJEF for 12 h were decreased in a dose-dependent manner, but levels of active forms such as cleaved caspase-3 and -9 increased in a dose-dependent manner.

### 3.6. Effect of OJEF on the Expression of Cell Cycle-Related Proteins

We performed western blotting to confirm the cell cycle arrest data from FACS analysis at the protein level. As shown in Figure 5(b), the expression levels of CDK2, CDK4, cyclin D1, and cyclin B1 diminished in MDA-MB-231 cells after treating with each concentration of OJEF for 12 h in a concentration-dependent manner.

### 3.7. Effect of OJEF on the Expression of Invasion and Metastasis-Related Proteins

We performed western blotting to confirm the effect of OJEF on various proteins involved in cancer cell metastasis. As shown in Figure 5(c), the expression amounts of claudin-1, zo-1, E-cadherin, *β*-catenin, integrin *β*1, and MMP-9 in MDA-MB-231 cells after treatment with each concentration of OJEF for 12 h decreased in a dose-dependent manner.

### 3.8. Effect of OJEF on the Expression of MAPKs-Related Proteins

We performed western blotting to identify the expression levels of the MAPK family proteins known to affect upstream signaling pathways. As shown in Figure 5(d), the expression levels of total p38, ERK, and JNK in MDA-MB-231 cells after treatment with each concentration of OJEF for 90 min (p38 and ERK) and 60 min (JNK) were not changed in a dose-dependent manner, but those of active phosphorylated forms such as p-p38 and p-ERK were increased in a dose-dependent manner.

## 4. Discussion

In this study, we conducted cell viability assay using WST, microscopic observation of chromatin condensation using DAPI staining, apoptosis analysis using Annexin V/PI staining, cell cycle arrest analysis using PI staining, and protein expression analysis by western blotting. The nuclear changes induced by OJEF were directly observed by confocal microscopy after DAPI staining. After 24 h of treatment with OJEF, chromatin condensation was increased by apoptosis in a dose-dependent manner and apoptotic bodies with varying sizes formed, significantly more than that observed in the control (Figure 2(a)). To detect early and late apoptosis in MDA-MB-231 cells added with OJEF, FACS was performed after Annexin V/PI staining. As shown in Figures 2(b) and 2(c), the late apoptosis rate (26.17%) at 60 *μ*g/mL of OJEF was higher than that of the control (10.66%). Treatment with OJEF increased the total apoptosis rate and decreased the survival rate. Besides, as determined by FACS analysis, OJEF increased the sub-G1 phase cell population in a dose-dependent manner (Figure 3). These results suggest that OJEF may not only induce apoptosis but also induce cell cycle arrest at the G1/S phase in MDA-MB-231 cells. Additionally, protein expression analysis by western blotting was performed to examine apoptosis, cell cycle arrest, and antimetastasis in order to investigate each mechanism at a molecular level [17, 18, 22–26]. First, induction of apoptosis mediated through the intrinsic pathway involving caspase-3 and -9 and bcl-2 [27–29]; the extrinsic pathway involving caspase-3 and -8 [29, 30]; and the ER stress response pathway involving caspase-3, -9, and -12 [31, 32] by OJEF was all elucidated (Figure 5(a)). Thus, the antibreast cancer mechanism of OJEF in MDA-MB-231 cells is regarded to be attributed to the close cooperative interaction of 3 different pathways of apoptosis [27–32]. However, unlike previous studies in gastric cancer cells, apoptosis through the p53-mediated pathway was not observed in this study [3, 4]. Second, the induction of cell cycle arrest in G1/S checkpoint by OJEF was clearly observed in MDA-MB-231 cells in this study as shown in Figures 3 and 5(b). This suggests that cell cycle arrest occurs in G1/S via CDK2, CDK4, and cyclin D1 and the cell proliferation is inhibited by OJEF. The results of cell cycle arrest at the G1/S phase by OJEF observed with FACS analysis (Figure 3) were well consistent with the data obtained from western blotting (Figure 5(b)) because downregulation of CDK2, CDK4, and cyclin D1 suggests cell cycle arrest at the G1/S phase. Meanwhile, downregulation of CDK2 and cyclin B1 (Figure 5(b)), which are known to be associated with cell cycle arrest at the G2/M phase was inconsistent with FACS analysis (Figure 3), because the G2/M phase occurs later than the G1/S phase, which is arrested first by the OJEF. Third, antimetastasis of estrogen-independent MDA-MB-231 cells known to be prone to metastasize by OJEF was well confirmed through observing downregulation of many proteins involved in metastasis including tight junction, adherens junction, invasion, and migration as well as wound healing assay (Figures 4 and 5(c)). Specifically, claudin-1, zo-1, E-cadherin, and *β*-catenin associated with tight junction, E-cadherin and *β*-catenin associated with adherens junction, integrin *β*1 and MMP-9 associated with invasion, and E-cadherin, integrin *β*1, and MMP-9 associated with collective migration of cancer cells were all downregulated by OJEF [33–35]. Although the Wnt protein was not identified, levels of its effector product for metastasis, *β*-catenin, were decreased, indicating that metastatic processes including invasion and migration were inhibited [36, 37]. Moreover, MAPK family-mediated upstream signal transduction through p-p38 and p-ERK is considered to affect the downstream signal transduction including induction of apoptosis, cell cycle arrest, and antimetastasis [38–42] although we could not confirm the regulation mechanism by p-JNK.

## 5. Conclusion

We have executed an integrated study on the anticancer effect of OJEF, which extensively induced apoptosis, arrest of cell cycle, and antimetastasis on estrogen-independent MDA-MB-231 human breast cancer cells known to be prone to metastasize.

## Data Availability

The data used to support the findings of this study are available from the corresponding author upon request.
